# Synthesis and cell-free cloning of DNA libraries using programmable microfluidics

**DOI:** 10.1093/nar/gkv1087

**Published:** 2015-10-19

**Authors:** Tuval Ben Yehezkel, Arnaud Rival, Ofir Raz, Rafael Cohen, Zipora Marx, Miguel Camara, Jean-Frédéric Dubern, Birgit Koch, Stephan Heeb, Natalio Krasnogor, Cyril Delattre, Ehud Shapiro

**Affiliations:** 1 Applied Mathematics and Computer Science and Biological Chemistry, Weizmann institute of science, Rehovot, Israel; 2 Illumina Inc., Grenoble, France; 3 Centre for Bio-molecular Sciences, University of Nottingham, Nottingham, UK; 4 School of Computing Science, Claremont Tower, Newcastle University, Newcastle, UK

## Abstract

Microfluidics may revolutionize our ability to write synthetic DNA by addressing several fundamental limitations associated with generating novel genetic constructs. Here we report the first *de novo* synthesis and cell-free cloning of custom DNA libraries in sub-microliter reaction droplets using programmable digital microfluidics. Specifically, we developed Programmable Order Polymerization (POP), Microfluidic Combinatorial Assembly of DNA (M-CAD) and Microfluidic In-vitro Cloning (MIC) and applied them to *de novo* synthesis, combinatorial assembly and cell-free cloning of genes, respectively. Proof-of-concept for these methods was demonstrated by programming an autonomous microfluidic system to construct and clone libraries of yeast ribosome binding sites and bacterial Azurine, which were then retrieved in individual droplets and validated. The ability to rapidly and robustly generate designer DNA molecules in an autonomous manner should have wide application in biological research and development.

## INTRODUCTION

The study of biology has accelerated to an unprecedented pace due to the development of methods for massively parallel cell-free cloning and DNA sequencing and their integration into miniaturized hardware in desktop next generation sequencing (NGS) machines (1).

Conversely, the engineering of biology is still largely restrained by fundamental limitations of gene synthesis and cloning methodologies (2–5). The development of novel approaches and apparatus for gene synthesis and cloning and their integration into desktop devices is positioned to improve our ability to write genetic code to the extent that NGS technology improved our ability to read it. Specifically, microfluidics is positioned to improve our ability to write custom DNA molecules by increasing the throughput of gene synthesis through parallelization, reducing its cost through miniaturization and simplifying complex synthesis through autonomous liquid handling. The development of novel methods and hardware for microfluidic gene synthesis is the subject of intensive ongoing research. Specifically, several studies have already shown that genes can be made *de novo* using various microfluidic technologies that run one-pot enzymatic gene synthesis methodology (6–8).

However, for microfluidic gene synthesis to advance it should overcome the limitation of strictly assembling genes in one pot reactions (6–8) and accommodate a wider range of assembly methods, despite the fact that they require more complex liquid handling schemes. These could be realized by programmable microfluidic platforms that execute *ad hoc* microfluidic gene synthesis methodologies. Crucially, advanced microfluidic platforms for gene synthesis must have programmability over droplet routing. This would enable the implementation of complex, parallelized liquid handling schemes which are challenging to execute both manually and on liquid handling robots and offer the flexibility required to accommodate different construction protocols.

Programmable microfluidic platforms open opportunities for developing novel *ad hoc* gene synthesis methodologies that utilize the advantages of programmability. However, these have not been developed, primarily due to programmability limitations of microfluidic technologies used for gene synthesis to date—specifically in supporting random access, complex droplet routing schemes that are often required in more advanced, hierarchical gene synthesis methods (9–11). To this end, we demonstrate how complex gene synthesis schemes associated with the parallel hierarchical DNA library synthesis (9–11) of multiple rationally designed variants, each made and retrieved individually, can be accomplished through programmable microfluidic droplet routing technology (see M-CAD section), effectively overcoming the one-pot assembly barrier of existing microfluidic gene synthesis (6–8). Additionally, we developed an *ad hoc* Digital Micro Fluidic (DMF) gene synthesis method for *de novo* DNA construction from oligonucleotides that utilizes droplet routing programmability (see POP assembly).

Cloning of synthetic constructs is the second and final phase in the pipeline of generating synthetic genetic material. Despite the development of new cloning approaches it is still a major bottleneck in gene synthesis and remains largely un-addressed with microfluidics. Instead, microfluidic gene synthesis typically resorts to traditional cloning of the assembled genes into bacteria off-chip (12,13). Only recently has electroporation-mediated transformation of bacteria and yeast been demonstrated using microfluidics for the first time (14). However, fully harnessing the potential of microfluidic gene synthesis will require the development of on-chip methods for cell-free cloning of DNA constructs that bypass the need for bacterial/yeast transformation. These could, theoretically, have throughput similar to that of NGS cell-free cloning. However, since gene synthesis and cloning are preparative rather than analytical procedures, they (in contrast to NGS cell-free cloning) must also enable the physical retrieval of cloned DNA molecules in individual droplets. Cell-free cloning of synthetic genes was first accomplished by us (15) and later by others with higher throughput (16–18). However, it has not yet been accomplished using microfluidic technology. Here we demonstrate the first microfluidic cell-free cloning of synthetic genes, including retrieval and validation off-chip. Applying microfluidic technology for cloning and gene synthesis remains a major untapped source of innovation in synthetic biology and bio-engineering. Electro-Wetting On Dielectric (EWOD) technology, also known as DMF, has been demonstrated in the past to offer the programmability required to implement molecular biology protocols (19–22), but has not been applied to the controlled synthesis and cloning of designer genes and their libraries. In this manuscript we present a programmable DMF device and the accompanying *ad hoc* methodologies for (i) synthesis of genes *de novo* (POP assembly), (ii) construction of rationally designed combinatorial DNA libraries with each variant individually retrieved (M-CAD) and (iii) cell-free cloning of assembled synthetic DNA. The methods were executed using a desktop DMF device and the resulting DNA molecules produced were inspected and validated both at the sequence level using cloning and DNA sequencing and for functionality downstream.

## MATERIALS AND METHODS

### DMF DNA construction

#### POP assembly

Each assembly droplet is 0.3 μl and contains at each phase of the assembly process: a pair of the correct (different pair for each phase of construction) external overlap extension primers (0.1 pmol each), 1 fmol of the correct template molecule template (different template molecule for each phase of construction), 1X hot start KOD buffer, 0.02U KOD Hot Start enzyme, 200 μM of dNTP.

EWOD Cartridge Thermal Cycler program: Enzyme activation at 95°C for 10 min, 4 cycles of thermal cycling per each primer pair and template (denaturation 95°C 5 s, annealing at *T*_m_ of primers 5 s, extension 72°C 15 s/kb) with 64X dilution between POP assembly phases. The purpose of the dilution between stages of POP assembly is to dilute out the primers from the previous POP stage and, as a result, avoid a purification step between POP stages.

POP assembly programs were uploaded to the instrument and ran once cartridges were filled with the filler fluid, loaded with the POP reagents at the appropriate wells and the cartridge placed inside the device. Surfactant was added to the POP assembly reactions on cartridge to avoid the formation of stationary droplets. POP Assembly products were eluted manually from the dedicated collection wells.

The program used for POP assembly droplet processing (POP.ade) can be downloaded and used to execute POP assembly on commercially available instruments.

#### Single molecule polymerase chain reaction (smPCR)-based cell-free cloning

smPCR was performed with KOD hot start (Novagen) polymerase on the EWOD cartridge. Single molecule templates were obtained via limiting dilution and amplified through shuttling of droplets between temperature zones. smPCR reactions were performed in various volumes on cartridge ranging between 0.3 μl and 1.2 μl final volume. Primers containing only CA bases (no GT) were used for smPCR amplification to avoid primer dimer formation. Each (0.3–1.2 μl) reaction droplet Reaction contains: 1X hot start KOD buffer, 0.02U-0.08U KOD Hot Start enzyme, 0.1–0.4 pmol of the CA primer, 200 μM of dNTP.

EWOD Cartridge Thermal Cycler program: Enzyme activation at 95°C for 10 min, denaturation 95°C 5 s, annealing at *T*_m_ of primers 5 s, extension 72°C 15 s/kb, 50 cycles. It is important that the PCR is prepared in sterile environment using sterile equipment and uncontaminated reagents. POP assembly programs were uploaded to the instrument and ran once cartridges were filled with the filler fluid, loaded with the POP reagents at the appropriate wells and the cartridge placed inside the device. Surfactant was added to the POP assembly reactions on cartridge to avoid the formation of stationary droplets. smPCR products were eluted manually from the dedicated collection wells.

The program used for POP assembly droplet processing (POP.ade) can be downloaded and used to execute POP assembly on commercially available instruments.

#### M-CAD –microfluidic combinatorial assembly of DNA

M-CAD was performed with KOD hot start (Novagen) polymerase on the EWOD cartridge. DNA templates used for generating the library building blocks on cartridge were plasmids purified from bacteria. Overlapping PCR building blocks were assembled using thermal cycling as follows: EWOD Cartridge Thermal Cycler program: denaturation 95°C 5 s, annealing at *T*_m_ of primers −5, extension 72°C 15 s/kb, 10 cycles. Each 300 nl reaction droplet Reaction contains: 1X hot start KOD buffer, 0.02U-0.08U KOD Hot Start enzyme, two overlapping PCR building blocks and 200 μM of dNTP.

The Assembled is then serially diluted on-cartridge and PCR amplified with external primers using EWOD Cartridge Thermal Cycler program: denaturation 95°C 5 s, annealing at *T*_m_ of primers −5, extension 72°C 15 s/kb, 30 cycles. M-CAD assembly programs were uploaded to the instrument and ran once cartridges were filled with the filler fluid, loaded with the M-CAD reagents (tempaltes, primers, polymerase mix) at the appropriate wells and the cartridge placed inside the device. Surfactant was added to the M-CAD assembly reactions on cartridge to avoid the formation of stationary droplets. Assembled target molecules of the Azurin combinatorial library were eluted manually from the dedicated collection wells and verified by sequencing.

The program used for POP assembly droplet processing (POP.ade) can be downloaded and used to execute POP assembly on commercially available instruments.

#### Azurin library western blot

For detection of Azurin by western blot, Pseudomonas aeruginosa PAO1 wild type and azurin in frame deletion mutant (published elsewhere) were grown in 10 ml LB (Luria Bertani) broth for 16h under vigorous shaking (200 rpm). A volume of 1 ml culture was centrifuged and cell pellets suspended in sodium dodecyl sulphate (SDS) sample buffer containing 6% β-mercaptoethanol. Samples were run on 30% SDS-polyacrylamide gel and blotted to Hybond-ECL nitrocellulose membrane (Amersham) in a BioRad Mini-Protean system. The blot buffer consisted of (12.5 mM) Tris-Base, 96 mM glycine and 20% methanol. After blotting the membrane was incubated overnight at 4oC in TBST (1 mM Tris-HCl, 150 mM NaCl, 0.05% Tween 20, pH 7.4) containing 5% milk (from non-fat milk powder) and azurin antibody (1:2000) (Yamada et al., 2005). The membrane was washed once with TBST and incubated for a further 60 min at room temperature with TBST containing anti-goat IgG alkaline phosphatase conjugate (1:5000). Azurin was detected using ECL chemiluminescence detection kit (Amersham). Commercial azurin (Sigma) was used as a standard.

#### DMF protocol validation

A validation process is applied for each protocol before it is routinely used. Droplets typically fail to move because of improper surface tension and viscosity with respect to voltage applied. Each reagent in a protocol is made to be compatible with the voltage sent: surfactant concentration is adjusted to enable correct droplets operations such as dispenses or moves. Electrode actuation sequence, i.e. timing between each actuation/de-actuation of the electrodes, is also of importance, and we experimentally determine the right timing before locking down a protocol. Electrode failure never occurred for the timescale of the experiments. Once a protocol is validated the frequency of droplet failure is essentially zero.

##### Transformations to yeast

The POP-generated variants were transformed into the yeast master strain using the LiAc/SS carrier DNA/PEG method. Cells were plated on solid agar SD-URA selective media and incubated at 30°C for 3–4 days. Transformant colonies were handpicked and patched on SD-URA + NAT (Werner BioAgents) agar plates. Correct transformation was verified for all variants by PCR amplification from the yeast's genome, gel electrophoresis and verified by sequencing.

#### Yeast gene expression measurements

POP library strains were arrayed on SD-URA+NAT agar plates in 96 colony format using a robotic colony arrayer (RoToR, Singer instruments). The colony arrayer was used to inoculate the library into SD-URA in 96 well microplates (Greiner bio-one, 781162). Following over-night incubation, strains were diluted 1:20 into SD complete media and cultured. A microplate reader (Neotec Infinite M200 monochromator) was set to measure growth (Absorbance at 600 nm), mCherry (E.x. 570 E.m. 630) and YFP expression (E.x. 500 E.m. 540) in 10 min intervals. Each cycle contained 4 min of orbital shaking at amplitude of 3 mm. The number of cycles was set to 100 (16h) and the temperature to 30°C. The entire procedure was performed in three times.

The library transformation master strain was generously provided by the lab of Prof. Eran Segal. The master strain was created by integrating into the yeast genome a cassette expressing the fluorescent mCherry under a TEF2 promoter and a promoter-less YFP, followed by a NAT (Nourseothricin) resistance marker under its own promoter. The entire sequence was inserted into the his3Δ1 locus of strain Y8205 (A strain derived from *S. cerevisiae* S288C, BY4741, mat alpha, Charlie Boone lab).

A microplate reader (Neotec Infinite M200 monochromator) was then set to measure the following parameters in cycles of 10 min: Cell growth (as extracted from absorbance at 600 nm) and YFP expression (Ex. 500 Em. 540). Each cycle contained 4 min of orbital shaking at amplitude of 3 mm. The number of cycles was set to 100 (16h) and the temperature to 30°C.

#### DNA sequencing

Sequencing of the POP DNA library and the smPCR products was performed standard Sanger sequencing at the Weizmann institute sequencing facility.

#### DNA oligos

All DNA oligos for POP assembly were ordered as Ultramers from Integrated DNA technologies (IDT). Oligos for smPCR and for the construction of the Azurin library were ordered as standard desalted oligos from IDT.

#### DNA oligo design

The DNA oligos used for POP assembly were designed to have a minimal *T*_m_ of 60 degrees to the oligos of the subsequent POP step (so that optimization of annealing temperature is possible) and to be under 120 bp in length (since Ultramers longer than 120 bp do not function as good primers in our experience). All primer sequences are available from the supplementary file, pages 11–12.

## RESULTS

### Digital microfluidics system

We designed a microfluidic desktop device that assembles synthetic genes and clones them using a combination of existing and *ad hoc* methodologies. The device uses electro-wetting on dielectric (EWOD) technology, also known as DMF, to manipulate in parallel multiple 300nl droplets in a programmable manner by the application of an electric field under direct software control (23–28). LH operations such as dispense, transport, split and merge are combined to implement complex LH protocols without the use of pumps, valves or moving parts.

**Figure 1.**
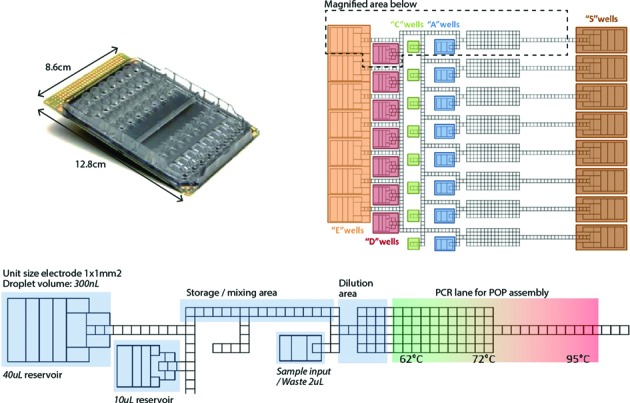
Overview of DMF cartridge design. Top: dimensions and general layout of the cartridge, with annotated reservoirs names. Bottom (magnified): annotated schematics of a section of the microfluidics cartridge layout. Different regions corresponding to different functions are highlighted. Dilution buffer reservoir contains the reagent used to perform dilutions to reach single molecule DNA concentrations/ PCR Mix. Other reservoirs are used to hold master mixes that contain the appropriate pairs of primers. During each phase of the DMF program the storage area is continually loaded with the droplets required for the following stages of the run to speed up the protocols. The dilution zone is used to perform the serial dilutions for POP assembly, M-CAD and smPCR based *in vitro* cloning. The PCR lane consist of three temperatures zones at 62°C, 72°C and 95°C. Temperature ramping is accomplished by DMF-based droplet shuttling between temperature zones.

The system (Figure 1) consists of (i) a DMF cartridge on which liquid droplets are shuttled and reactions takes place (Figure 1, top left) and (ii) a microfluidics apparatus into which the cartridge is mounted that embeds the electronics for generating the EWOD phenomenon.

The cartridge consists of two parts: (i) a printed circuit board bottom plate and (ii) a plastic injected-molded cover plate where througholes are used as wells for liquid loading into the working area of cartridge. The core of the cartridge, the PCB, comprises typical material stack used in electrowetting devices, i.e. a conductive layer defining the electrodes, a dielectric layer used to build up the capacitive force and a hydrophobic coating that exerts a contact angle of 106° in air, of 160° in the filler fluid without voltage and 60° at maximum voltage applied for water with 0.01% Tween 20. Tween 20 at 0.01–0.05% final concentrations facilitates robust droplet routing and is also an integral part of standard DNA polymerase buffers and therefore does not interfere with DNA polymerase-based assembly methods. The voltage used was 200–300V and the frequency used was 10–1000 Hz. Current stack and voltage provide to the droplet a typical capacitive energy of 0.039J.m-2. The cartridge contains a gap space of 300um between the cover plates and is filled with 5 ml of 5cSt silicone oil filler fluid, which facilitates robust droplet transport and reduces evaporation during temperature cycling. The electrode structure layout of the cartridge is defined in Figure 1 (top right) and can also be seen in Video's S1–S3. We used the same DMF cartridge layout (Figure 1, top right) to perform both DNA assembly methods and *in vitro* cloning. The distribution of througholes in the cartridge plastic injected-molded cover are depicted as A, C, D and E wells (each containing 7–8 wells) in Figure 1 and Videos 1–3. These througholes (30 in total) are used as input/output channels for manual loading using a standard manual micropipette to the hydrophobic-coated bottom plate (see Video S4 for loading and retrieving reagents from the cartridge). The operational volumes of these reservoirs range from 2 μl to 40 μl, with the dead volume varying between 0% and 10% of the total input depending on the liquid properties. Manual collection of droplets containing the assembled products was performed using 10 μl pipette tips and was visually guided by the merging of droplets with dyed droplets prior to their transport to the collection well where they are retrieved. The AC voltage sent to each electrode is provided by a dedicated instrument from Illumina, consisting of an electro-wetting effector instead of using traditional voltage generators and relays (25). Additionally, three independent heater bars located at defined zones (see Figure 1) directly underneath the cartridge are used to perform reactions (such as PCR) by shuttling the droplets between the different temperature zones (Figure 1, bottom right). Temperature calibration of the heater bars and the gradient between them is performed once and for all at the instrument reception using miniature thermocouples (150um thick, Thin Leaf-Type Thermocouples, 88309, Omega Engineering Inc.) inserted between the PCB board and the plastic top plate inside the filler fluid using the open through hole of the top plate. The heater bars set-points were calculated to reach the needed temperature inside the cartridge (the set-points are therefore higher than the measured temperature, i.e. 60C, 72C, 95C). Temperature decreases gradually between the different zones inside the cartridge without going below the targeted temperature. Compared to IR temperature measurement this method provides a more accurate account of droplet temperatures since it is not possible to image the PCB board with an IR camera when the cartridge is filled with silicone oil. By measuring the temperature with miniature thermocouples inside the filler fluid that surrounds the droplets, we obtained the most accurate indication of droplet temperature. Videos S1–S3 depicting POP, M-CAD and MIC were filmed using a Chameleon CMLN-13S2C-CS USB color camera (Point Grey Research) with a Xenoplan 23/1,4 magnification lens (Schneider-Kreuznach).

### Programmable order polymerization (POP) assembly

We set out to test whether control over droplet maneuvering with EWOD enables the implementation of complex microfluidic LH schemes for gene synthesis. We designed POP DNA synthesis, an *ad hoc de novo* gene synthesis method specifically tailored for DMF. We show how a synthetic library of YFP 5' UTR variants can be autonomously built *de novo* using POP assembly on-chip. We then demonstrate how these synthetic YFP variants can be cloned *in vitro* on the same assembly chip using smPCR-based *in vitro* cloning and retrieved from the chip for downstream validation of the method using transformation, sequencing and gene expression measurement.

**Figure 2.**
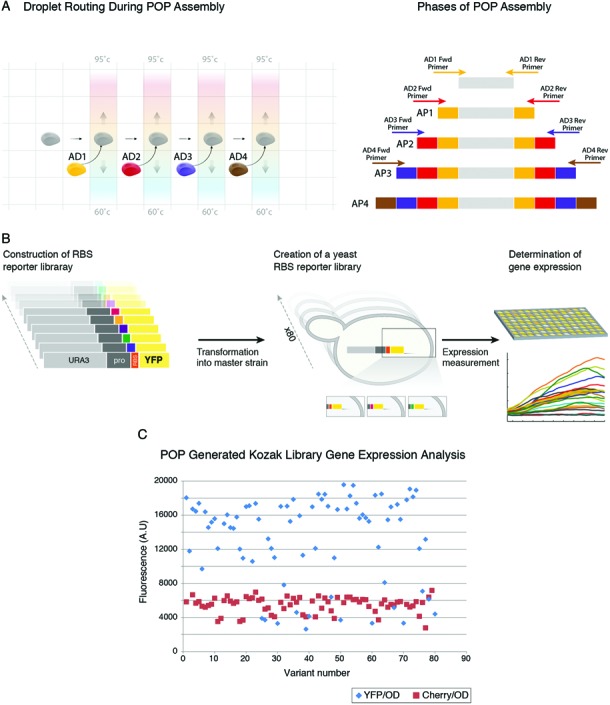
DNA Synthesis using DMF. (**A**) POP assembly schematics. A droplet containing template DNA (gray) is combined with assembly droplet 1 (AD1) that contains the primers and assembly mix to form a reaction droplet (thermo-cycled, in gray) in which assembly product 1 (AP 1) is generated. The AP1 containing droplet is then combined with assembly droplet 2 (AD2) that contains the primers and assembly mix to form a new reaction droplet (thermo-cycled, in gray) in which assembly product 2 (AP2) is generated. The process is iterated (with AD3, AD4, etc.) until the full length molecule (AP4) is constructed. (**B**) Schematic representation of the RBS DNA library, its transformation into yeast and its gene expression measurement in 96-well format using a plate reader. (**C**) Gene expression measurements of POP-generated 5′UTR library showing a 10-fold variation in gene expression across library variants. (**D**) Overview of M-CAD, a method that receives a set of DNA molecules as input and copies segments from them to create variants according to specification in a digital microfluidic device. A. M-CAD enables the implementation of the basic text editing operations on DNA molecules such as Cut, Copy, Paste, Cut and Paste, and Copy and Paste. B. The text editing operations required for constructing a DNA library from a set of DNA inputs (top of the tree) are translated into a tree in which vertices are DNA molecules and edges are the editing operations (cut, paste, etc.) for generating the vertices. Purple represents DNA that was originally present on the input DNA molecules and red represents synthetic DNA added during the editing process (from PCR primers for example). Vertices V1–V10 are the final 10 variant DNA molecules in this example of –CAD construction C. various input DNA molecules and primers are loaded onto the device and droplets generated from them (colored droplets) are routed on cartridge to assemble the target DNA molecules in a pre-defined manner (as determined in B).

Specifically, in POP assembly (Figure 2A) a synthetic construct is built inside–out from an initial DNA template (which can be either natural or synthetic) via an ordered set of serial elongation reactions. In each of the elongation reactions only two DNA oligos extend the synthetic dsDNA construct from both its termini through several cycles of polymerase-based overlap-extension. Once a pair (for example, AD1 in Figure 2) of oligos has completed extending the construct a fresh droplet containing the next specific oligo pair (in this case AD2 in Figure 2) is programmed to merge into the assembly reaction. Each oligo pair uses the extensions created by the former pair as hybridization sites and further extends the construct inside-out using overlap extension. Specifically, primers from Assembly Droplet 2 (AD2) use hybridization sites created by primers from AD1, primers from AD3 use hybridization sites created by primers from AD2 and so on. This process of temporally ordered, distinct overlap extension events is iterated, each time using the correct assembly droplet until the full length molecule is constructed. The process depends on the accurate integration of specific assembly droplets into the POP assembly reaction at specific times on chip and is completely pre-programmed.

In contrast to one-pot gene synthesis methods carried out in general (29,30) and specifically in microfluidic devices (6–8), POP assembly substantially reduces the complexity and increases reliability of the assembly reaction. It simplifies the assembly by reducing to the minimum the number of components that are simultaneously assembled, enabling individual reaction condition optimization for each DNA component in the system. This is in contrast to one-pot assembly methods that face the problem of simultaneously optimizing specificity and reaction conditions for multiple DNA components (6–8). Additionally, one-pot assembly methods face the problem of miss-hybridization between the multiple DNA components present in the reaction. This complexity is often mitigated through various computational and biophysical methodologies aimed at controlling the correct order and hybridization specificity between multiple oligos that exist in a single reaction (30). However, avoiding cross-hybridizations when multiple DNA fragments are mixed and amplified in a single assembly reaction has proven a challenge and one-pot assembly remains prone to construction failure, often resulting in labor-intensive sifting through multiple clones that harbor non-specific assembly products. POP assembly using DMF mitigates this problem by integrating a dilution step between each assembly phase that effectively reduces the concentration of previous assembly components that are no longer required in the reaction. This is followed by a specific increase in the concentration of the components that are required specifically for the next stage. This fine control over the components of the assembly reaction at every stage of the construction process reduces the generation of off-track assembly products. Physical and temporal separation between assembly components relieves computational design constraints on the oligos sequences, enabling us to use simple design rules for the oligo components. We set the DMF system to implement POP assembly protocols for the *de novo* construction of a YFP reporter gene fused to a 5′ UTR library (Video S1). For comparative purposes, we compared the POP assembly of the YFP library head-to-head to a typical one pot-assembly reaction using Gibson assembly (31) of the same YFP molecule. Gibson assembly resulted in an increasing fraction of spurious assembly reactions with an increase in the number of DNA fragments (7 in total) in the assembly reaction (Supplementary Figure S1, from left to right). In contrast, POP assembly showed almost no spurious assembly products in any of its construction stages (Supplementary Figures S2–S5), starting from POP assembly level 1 (Supplementary Figure S2) and up to the final POP assembly level 4 (Supplementary Figure S5). DMF POP assembly used nine Ultramer (IDT DNA) oligonucleotides as building blocks in total and proceeded according to the principles of POP assembly, with each POP stage adding approximately 160 bp of synthetic DNA to the construct. Each step in the assembly process was monitored separately using gel electrophoresis to verify its success (Supplementary Figures S2–S5). The complete POP assembly plan and its execution are shown in full detail in Video S1 (simulation) and Video S1, respectively. Following assembly, 300 nl droplets containing the final YFP ribosome binding site (RBS) library were combined on-cartridge with a dyed 300 nl droplet to ease manual collection of the droplet from the elution well. The DNA was amplified off-cartridge with external primers to provide sufficient DNA for downstream processing. The POP-generated DNA library was fused using previously developed methods (9,32) off-cartridge with a URA selection marker copied from a yeast intron library (33) to yield a 2.5 Kb fragment (Figure 2B, left). We transformed this construct into the genome of a *Saccharomyces cerevisiae* (*S. cerevisiae*) master strain using homologous recombination downstream of a TEF promoter at the his3Δ1 locus and recorded their YFP gene expression (see methods). The sequence of POP assembly constructs was validated from 56 clones by amplification of the corresponding part of the genome and Sanger sequencing (see supplementary material for representative Sanger sequencing data). The POP sequencing error-rate was 1/450 and was determined by dividing the total number of errors (22), all of which were substitutions, by the total number of sequenced bp (9710 bp). This error-rate is expected and fits nicely within the range of error-rates widely reported in the literature for *de novo* DNA synthesis methods (8–18) and is also similar to the error-rate that we obtained using other *de novo* DNA synthesis methods in our lab (9,15)., demonstrating that the use of DMF technology does not introduce unexpected error into the DNA construction process. To test whether POP assembly interfered in any way in generating library variability we tested whether the randomized 14 bases we inserted at the 5′UTR ribosome binding site (RBS) directly upstream of the ATG translation start site were maintained throughout the construction process and onto the yeast clones that were generated from them. Our results show that none of the clones that we sequenced (we sequenced 56 clones) had the same sequence (Supplementary Figure S6), indicating that, if POP assembly interferes with generating diversity it is to a very small extent. To validate the functionality of POP generated construct we also analyzed the variants gene expression profiles in yeast (detailed later in the manuscript). The time required to complete a parallel POP assembly of 8 individually retrievable genes of this length is approximately 1.5 h using the current cartridge design, with the limiting factor being the number of reservoirs for loading and storing reagents. Assembly throughput increases when constructing combinatorial DNA libraries since reagents are shared between different constructs. For example, the number of final targets can be increased if different target constructs share DNA building blocks between them, as in the case of the Azurine library (Supplementary Figures S7–S9) and as is often the case when constructing variants of a target molecule. A single cartridge run enables the construction of approximately 30 individually retrievable combinatorial constructs made from nine plasmids and a set of corresponding primers shared between library members (as in the Azurine library assembly below) within 3 h. Once the assembly is completed the cartridge can be re-used for additional runs of POP assembly (we have tried up to three runs on the same cartridge). POP assembly enables the *de novo* production of eight genes every 2 h (including loading and unloading time), with the limiting factor being the number of input and storage reservoirs for reagents required for construction.

### Validating the integrity of the POP-generated DNA library

In order to add a second layer of validation to the construction of the POP-generated YFP variants we planned the reporters to have completely randomized RBS and verified RBS diversity through DNA sequencing (Supplementary Figure S6). Comparison of Hamming distance between the RBS sequences of every library clone to all other library clones (see Supplementary Figure S11 for one specific but typical comparison) showed that each RBS clone is very different from all other library clones, indicating that POP assembly was able to capture a high degree of diversity in the RBS. We went on to transform the library into the genome of *S. cerevisiae* (see methods) downstream of a TEF promoter at the his3Δ1 genomic locus. To validate that POP YFP variant reporter clones are functional we measured their YFP fluorescence output using a 96-well plate reader (Tecan Infinite) in two independent duplicates which correlated very well (Supplementary Figure S12). The resulting YFP gene expression of the POP-generated reporter strains varied over 10-fold (Figure 2C), further validating the heterogeneity of the library.

While the focus of this work was to validate the construction methodology and not to study 3′ UTR regulation, see supplementary file S13 for additional downstream processing data and basic methodological validation of this library.

While POP assembly of the YFP variants as presented in this paper is a relatively small scale, proof of concept driven technological demonstration, it highlights the potential of using *ad hoc* microfluidic gene synthesis methodology for generating genetic material on demand. While it is useful for the study of synthetic genetic elements in general, it is most prominently useful for the study of rationally designed constructs that are required to be produced, retrieved and studied individually, in contrast to one-pot studies (34) which can be performed relatively easily without necessitating automation or computer aided design and manufacturing. To this end, we further set out to test whether microfluidics could be used for combinatorial gene synthesis and individual retrieval of rationally designed variants from existing plasmid templates (9–11) (in contrast to *de novo* synthesis demonstrated by POP assembly). This would enable for the first time the microfluidic synthesis and individual retrieval of rationally designed combinatorial variants that are made by copying DNA segments from different plasmids, assembling them into final constructs in different pre-determined combinations while editing their sequence with mutated oligos. To this end we implemented M-CAD, as discussed below.

### Microfluidic combinatorial assembly of DNA (M-CAD)

We developed procedures for M-CAD, which enable the individual construction of combinatorial, rationally designed DNA libraries and their retrieval as individual, separated variants from the cartridge (Supplementary Figure S8). We developed and validated M-CAD through the construction of a 24 variant library of the Pseudomonas aeruginosa azurin gene (azu). This library was rationally designed to explore the sequence of potential recognition sites for an RNA binding protein on the azurin mRNA coding region by altering its DNA\RNA sequence without altering its amino acid sequence. The basic building blocks of these rationally designed variants were available from a set of existing plasmids. They were amplified from the plasmids and assembled in different combinations (see Supplementary Figures S9 and S10) in a 2 step assembly process that also included performing PCR-based site directed mutations to these segments. In total, the microfluidic process was designed to construct 24 individually constructed and retrievable variants of the Azurin gene from a set of primers and plasmids.

To develop M-CAD we exploited our control over droplet manipulation in the DMF system to implement a complex microfluidic DNA assembly process, involving (i) the combinatorial re-use of DNA reagents in the assembly process, (ii) parallel multi-stage, multi-target DNA construction, (iii) utilization of multiple DNA inputs into the construction process, (iv) the development of on-cartridge, intermediate­ assembly-product storage strategies required for multi-stage DNA assembly schemes and (v) that the library variants can be accurately and individually eluted off the cartridge using simple manual pipetting operations with no detectible cross-target contamination. The complete protocol for the library assembly required 59 enzymatic reactions to run autonomously using a single DMF cartridge (see Supplementary Figure S14 for a summary of the design process). The 59 reactions were staggered in three successive assembly stages including up to 24 individual reaction droplets simultaneously. To facilitate throughput we developed a new droplet manipulation scheme dubbed ‘circular permutation PCR’ that enables PCR with multiple droplets on the same cartridge PCR lane. Shuttling of multiple droplets between temperature zones of a single PCR lane by using circular permutations (see video S2 and video S2 for its simulation), effectively triples (in this case) the throughput of each lane on DMF cartridges.

Hydrophobic proteins may, over time, foul the surface. However, the type of contamination that is problematic for DNA synthesis and cloning, as presented in this manuscript, is not protein contamination, but DNA contamination. Electrode tracks that pick up polymerase enzymes constituents from previous droplets do not pose a problem to the methods described in this manuscript since fresh droplets contain exactly the same polymerase. In contrast, DNA contamination could theoretically pose a problem, but DNA is hydrophilic and is widely acknowledged not to foul the hydrophobic coating of DMF cartridges. We cloned and sequenced 24 variants of the M-CAD library (which were all constructed together on the same cartridge) with no evidence of cross-contamination. Each of the M-CAD assemblies eluted individually off the cartridge had its expected variant sequence upon cloning and sequencing. If DNA cross contamination occurred we would expect at least one out of the 24 assemblies to produce a clone with the sequence of a contaminating variant. This result is expected due to the hydrophobic nature of the surface of the cartridge which is, if any, expected to be cross-contaminated with hydrophobic proteins, not hydrophilic DNA. We cannot rule out that trace amounts of DNA contamination may be present and could potentially be detected if deeper cloning and sequencing are performed. However, from the functional perspective of obtaining error-free variants of the 24 variants that we built (which is the focus of this method) DNA contamination did not pose a problem.

We used M-CAD to generate a library of 22 rationally designed variants of the Azurine gene by programming a DMF LH scheme that enabled the parallel assembly and individual retrieval of explicitly specified gene variants from the microfluidic cartridge. Assembly was accomplished through binary overlap extension reactions between PCR products. To design an appropriate DNA library construction scheme and the corresponding oligonucleotides used in the construction process we performed a shared component computational analysis of the Azurine library using a heuristic for maximizing DNA reuse in DNA library construction (35) and computational oligonucleotide design tools (9,32,36). These were also used for automatically generating a construction plan for the same library using a Tecan liquid handling robot controlled by a high level programming language (9). We translated this plan into two protocols for the construction of the library, one using liquid handling robots and the other using M-CAD (see Video S2 for simulation) and executed both. The M-CAD Azurine library construction process (Video S2) followed the aforementioned plan and consisted of PCR amplification of building blocks from three different plasmid templates using 6 different primers (Supplementary Figure S9), generating 12 PCR products (Supplementary Figure S9, blue nodes a and b). These 12 PCR products were further assembled in a combinatorial fashion using binary overlap extension to yield 24 out of the 27 possible target combinations and a final PCR amplification of the 24 full length assembled targets using external primers was also performed (see Video S3). All 24 library targets were then individually eluted from the cartridge, sized using gel electrophoresis following off-cartridge amplification (Supplementary Figure S10) and DNA sequenced to verify their sequence. Their sequence was identical to the same constructs made using a control construction of the same target molecules using liquid handling robot program (see supplementary sequencing file) and construction methodologies (27). To demonstrate that the method produced functional variants we analyzed one of the variants effect on the posttranscriptional control by an RNA binding protein. This was achieved by monitoring the production of the variant azurin protein in bacterial extracts by western blot analysis (Supplementary Figure S10). It shows that the mutant tested (which had relatively minor, synonymous alterations to an RNA binding site on the azurin mRNA coding region) was functional and had substantially altered azurin protein expression.

The sequence of 22 of the M-CAD variants was verified by cloning into E.coli and Sanger sequencing. In total 30 individual clones had to be sequenced to obtain a complete set of 22 clones with the correct, intended sequences. Once all the 22 variants were obtained error-free an additional 43 clones were sequenced in order to more accurately determine the error-rate. The sequencing results show an effective misincorporation rate of ≈6 × 10–4. This is calculated as follows: 73 (clones) x 477 bp = 34 821 bp; 19 errors in 34 821 bp is a frequency of 5.4 errors for 10 000 bp or 1/1832. The lower error-rate of M-CAD compared to POP is expected and in-line with the literature (32) since M-CAD is not a *de novo* DNA synthesis method which strictly uses synthetic oligos as building blocks. Instead, in addition to oligos M-CAD uses error-free plasmids as building block templates for synthesis, which reduce the error rate of the final construct.

Constructing and individually retrieving this 24 variant combinatorial azurin library using manual methods is not impossible, but would be a challenging effort due to its complexity—each of the 24 variants is made from a specific combination of 3–4 PCR fragments that are amplified from a specific subset of plasmids out of 9 different plasmids and requires the selection of specific sets mutating primers which are different for each fragment. In total it requires hundreds of liquid handling operations with a large set of fragment-specific reagents, which is challenging to execute manually. Constructing it using robotics is also possible but requires automation expertise and large scale liquid handling robots.

### Microfluidic in-vitro cloning (MIC)

**Figure 3.**
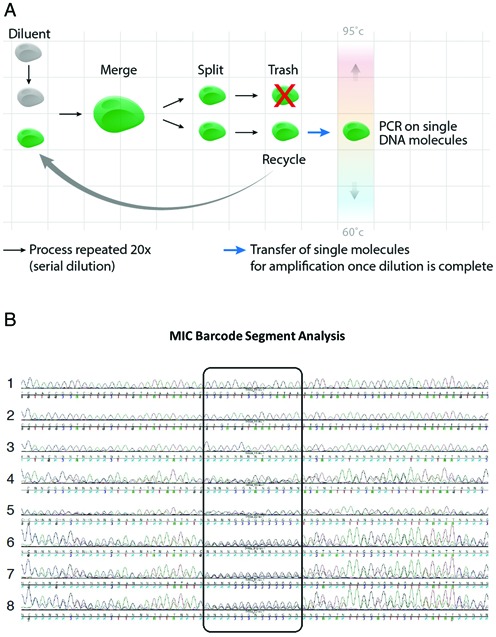
Scheme of cell free cloning using DMF. (**A**) The full length construct generated by POP assembly is subjected to DMF-based *in vitro* cloning using smPCR. A POP assembly product is iteratively diluted 2-fold using the following DMF operations: (i) merge (with diluent), (ii) split, (iii) trash (one half) and (iv) recycling of the second half back into the serial dilution. Once diluted, droplets are calculated to contain an average of one target DNA molecule per droplet and the single molecule droplets are programmed to travel to the PCR zone. In the PCR zone, single DNA molecule PCR droplets are amplified by PCR via their travel between the temperature zones. (**B**) Zoomed in view on the randomized 5′UTR region of a sequencing reaction from the DMF smPCR reactions. The sequencing chromatograms validate that single POP generated molecules were amplified on chip since the randomized 5′UTR sequence also functions as a barcode that verifies clonality (lanes 1–3). In contrast, negative control smPCR experiments (controls with many template molecules) show a clear pattern of variability in the 5′UTR/barcode region (center of chromatogram, lanes 4–8).

Once synthetic genes are made they are most often sequenced following cloning into bacteria or yeast, which is a major bottleneck for generating novel, error-free genetic material. To break this limitation methods for sequencing synthetic DNA clones without resorting to cellular cloning are needed (5,6). To this end, we utilized the same DMF cartridge design used for POP and M-CAD to clone the POP-generated DNA library using Microfluidic In-vitro Cloning (MIC) based on single-molecule PCR (see Video S3 for simulation). We employed a simple barcode region of consecutive *N* bases that, when Sanger sequenced, enables clear and simple verification of single molecules (see Figure 3B). The dilution of gene assembly products required to achieve single molecule concentrations is not known exactly (but is known approximately) in advance. Therefore, we employ an on cartridge serial dilution PCR that enables us to scan a large range of concentrations in one run of the microfluidic PCR, eventually resulting in at least one PCR that is clonal per construct.

We used the gold-standard method in validating that populations of *in vitro* generated DNA molecules originate from single DNA molecules by bar-coding the population of initial molecules with unique DNA barcodes. Using this method, if single molecules are amplified then their amplification products should all contain the same barcode sequence of the single molecule from which they all originated. If more than a single molecule was amplified in a reaction then the population amplified from them will have at least two different sequences at the barcode region. This methodology is widely accepted over multiple domains of molecular biology for validating single DNA molecules (see for example references (1,14,16) from this manuscript and many others in the field of cell-free cloning).

While DNA molecules may adhere to the surface following dilution, according to our sequencing results they are not amplified. Our dilution-based methods do not aim at generating single molecules per reaction chamber, but instead aim at generating dilutions that result in the subsequent amplification of single DNA molecules per reaction. It is often referred to as the effective dilution for single molecule amplification, not meaning that the amplified molecule was the only DNA template molecule present in the single molecule amplification reaction, just that it was the only one detectibly amplified. The reliability of this method has been extensively tested in multiple fields including, for example, next-generation-sequencing in which the same principle of DNA barcoding is used to validate clonality (single molecules).

To this end, we barcoded the DNA molecules with a combinatorial DNA code of 4^14^ options (14 N bases). Our results show (see Figure 3B) that MIC reactions that originated from a single DNA molecule show a single barcode sequence in Sanger sequencing (top three chromatograms). In contrast, MIC reactions in which we deliberately inserted more than a single molecule show multiple sequences in their chromatograms.

Specifically, we programmed a DMF protocol that performs a limiting serial dilution scheme of the POP assembly products to concentrations that lead to the amplification of single molecules. Diluted droplets calculated to produce amplification from single molecules are subsequently amplified, eight smPCR reactions at a time (Video S3). We retrieved the amplification products from the cartridge and Sanger sequenced their barcode region that contained 14 consecutive N bases, which enabled us to verify whether amplification indeed originated from single DNA molecules. The extreme variability of the barcode region (4^14^ optional sequences) ensures that if more than a single molecule is amplified in each reaction this would show up a heterogeneous bases in the sequencing chromatogram of that amplification. The sequencing results show that POP assembled single molecules were indeed amplified (Figure 3B, lanes 1–3) since they contained a single sequence at the barcode region. In contrast, negative control amplifications in which we included more than a single molecule per reaction showed the expected heterogeneous sequencing pattern at the barcode region (Figure 3B, lanes 6, 7, 8). Not all reactions calculated to contain concentrations that lead to the amplification of single molecules resulted in amplification products that originated from single molecules (Figure 3B, lanes 4 and 5). This is expected due to the natural distribution of the number of molecules per volume, even at concentrations that maximize the fraction of droplets with a single DNA molecule, as previously discussed by us (15). MIC provides a fast means for obtaining sequenced clones of synthetic, POP generated genes without resorting to cellular cloning and using the same microfluidic chip. False positive smPCR amplifications (reactions with more than one DNA molecule) can be kept to a minimum by working under dilution regimes of well under 1 molecule per PCR volume. Circular permutation PCR accommodates the required increase in reaction numbers and the low reaction volume (300nl) alleviates the cost associated with performing non-productive reactions (PCRs with no template DNA). Future work should also include Syber Green based real-time detection of which droplets PCR amplified and which do not so that only positive reaction are processed without the need for testing off-chip.

## DISCUSSION

Biological code is written, to a large extent, in DNA. Leapfrogging our ability to engineer this code, specifically within the realm of synthetic biology, depends on the development of reliable DNA construction biochemistry as well as efficient, reliable and programmable hardware to execute it. Our ability to read the DNA code has advanced considerably during the past decade due to the development and integration of biochemistry and machinery for DNA sequencing. Analogous machinery for rapid, in-­house prototyping of synthetic genes is positioned to revolutionize the engineering of biological systems. The development of methods that combine rapid *de novo* DNA synthesis with cell-free cloning using microfluidic technology, as presented in this manuscript, potentially paves a path for simple DNA synthesis and cloning machines that could widely broaden the access to synthetic genetic code. While our system improves on the parallelization, volume and programmability limits of current gene synthesis practice, it is currently limited by several factors, as discussed below. The maximal length of DNA assembly possible is not limited by DMF technology, but by the DNA construction biochemistry it executes. Our EWOD methods of synthesis are subject to the general length, efficiency and error rate limitations of PCR-based DNA construction methodologies. Specifically, the limitations of oligo and polymerase error-rate, which result in the classical exponential correlation between DNA length and the number of clones one needs to sequence to obtain an error free instance (15). Future work could involve the integration of enzymatic error correction methodologies (37). The throughput of gene synthesis using DMF technology presented in this work is limited by the specific cartridge hardware design and its feature sizes (300 nl droplets). An additional order of magnitude improvement on the throughput of our current cartridge and droplet size is feasible but would entail the design and fabrication of *ad hoc* DMF cartridges. Other droplet based technologies could potentially achieve even several orders of magnitude improvement in droplet size (such as Raindance), but do not enable the level of complexity and programmability required for the execution of the protocols presented here. To demonstrate the versatility and robustness of our methods we focused on producing libraries of constructs of different types, including both *de novo* DNA assembly and DNA assembly that is based on the modification of existing, natural DNA fragments. The libraries made had several key features: (i) target genes had pre-designed sequences, according to user specification, (ii) complex DNA library structure (Supplementary Figure S9, azurin) that is challenging for manual labor-based construction and (iii) construction strategy utilized two important concepts: (a) re-using fragments that are shared between target molecules and (b) maximizing the use of existing fragments as starting material for construction (Supplementary Figure S9). We went on to demonstrate DMF DNA construction by producing and sequence validating more than 70 distinct gene assemblies. Gel electrophoresis and DNA sequencing analysis over all assembled constructs show that spurious assembly products were rare (Supplementary Figures S1–S6) in our hands and sequencing errors are in-line with the expected error rates originating from the error rate of synthetic oligonucleotides and proof-reading polymerases used in the construction. The assemblies demonstrate EWOD can be used to execute challenging liquid handling schemes (Videos S1–S3), providing proof of principle for a sub-microliter scale, microfluidic device for DNA assembly on demand. The DNA construction methodologies described in the manuscript can be carried out manually, however, especially in the case of large DNA libraries, would be highly labor intensive, subject to complex liquid handling user errors, more expensive due to larger reaction volumes and not parallelized. For comparative purposes, the M-CAD DNA library was also constructed manually (it was not selected in advance for comparative reasons, but was a DNA library constructed in our lab for other purposes). In this comparison between EWOD and traditional manual gene synthesis reagent costs were reduced by a factor of 50 due to 50-fold reduction in reaction volume (0.3 μl) compared to typical manual pipetting reactions of 15 μl. Turn-around time was decreased by a factor of 10 due to three factors: (i) an improvement in parallelization of assembly reactions—in this demonstration 24 assembly reactions, each containing multiple reagents were performed in parallel. In our hands (a skilled DNA construction lab), one person cannot reliably perform more than 8 complex DNA assembly tasks in parallel. (ii) A 2-fold reduction in PCR-time due to rapid droplet routing between temperature zones, in contrast to the relatively slow temperature cycling of regular PCR machines. (iii) The avoidance of time-consuming manual steps required between steps of a multi-step protocol, such as: labeling of vials, writing protocols, and various operations related to the proper and reliable handling of plates and vials in complex protocols that involve the handling of many reagents and reactions. Future work should focus on prototyping *ad hoc* DMF cartridge designs dedicated and optimized for parallel construction of large DNA libraries and the possible integration of additional DNA construction biochemistries. Additional future work could demonstrate that the methods presented here are amenable to pairing with inexpensive chip-based oligo synthesis, which can be used as input template for POP assembly and/or M-CAD. Finally, cartridges were designed with standard micro-well plate and well dimensions in order to ease future work on interfacing of the cartridges with liquid handling robots for automated loading and retrieval of samples.

## SUPPLEMENTARY DATA

Supplementary Data are available at NAR Online.

SUPPLEMENTARY DATA
