# Supplementary material for: Synthesis and cell-free cloning of DNA libraries using programmable microfluidics
Source: Nucleic Acids Res. 2015 Oct 19;44(4):e35. doi: 10.1093/nar/gkv1087 (PMC4770201; doi:10.1093/nar/gkv1087)
Supplement: SUPPLEMENTARY DATA [file supp_44_4_e35__index.html]

Synthesis and cell-free cloning of DNA libraries using programmable microfluidics — SUPPLEMENTARY DATA 

# Synthesis and cell-free cloning of DNA libraries using programmable microfluidics

## SUPPLEMENTARY DATA

- SUPPLEMENTARY DATA
- SUPPLEMENTARY DATA
- SUPPLEMENTARY DATA
- SUPPLEMENTARY DATA
- SUPPLEMENTARY DATA
- SUPPLEMENTARY DATA
- SUPPLEMENTARY DATA
- SUPPLEMENTARY DATA
- SUPPLEMENTARY DATA
- SUPPLEMENTARY DATA
- SUPPLEMENTARY DATA
- SUPPLEMENTARY DATA
- SUPPLEMENTARY DATA
- SUPPLEMENTARY DATA
