# Supplementary material for: Synthesis and cell-free cloning of DNA libraries using programmable microfluidics
Source: Nucleic Acids Res. 2015 Oct 19;44(4):e35. doi: 10.1093/nar/gkv1087 (PMC4770201; doi:10.1093/nar/gkv1087)
Supplement: SUPPLEMENTARY DATA [file supp_gkv1087_nar-00880-met-k-2015-File008.pdf]

Supplementary Material

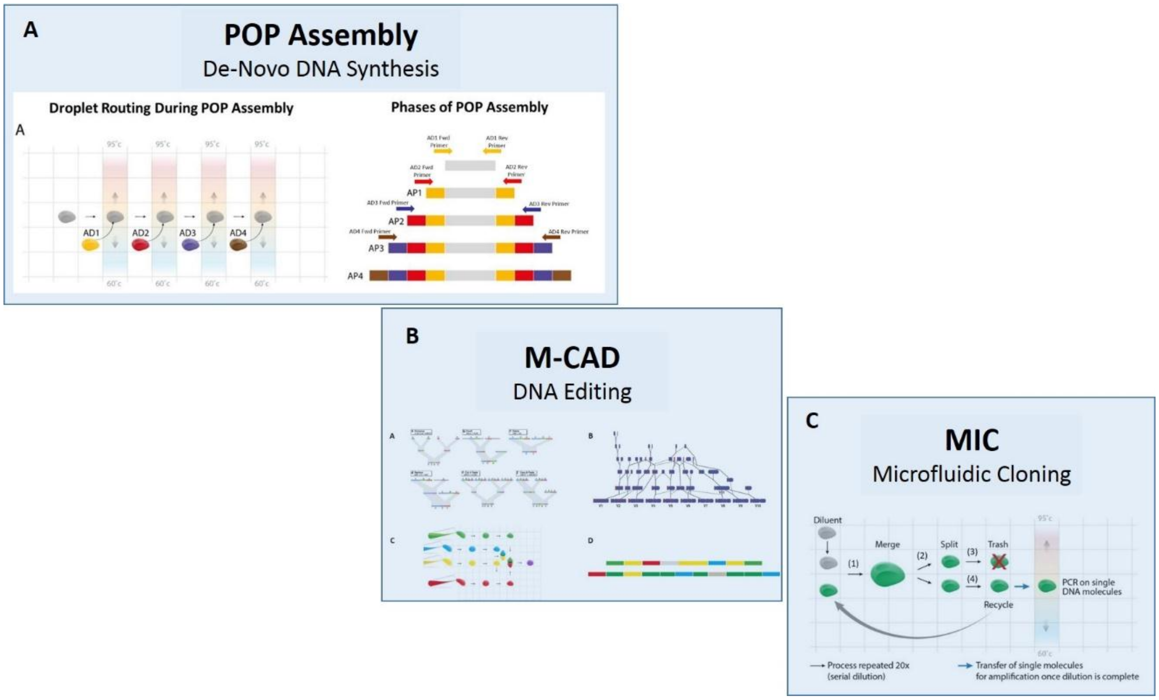

Figure S0 – overview of the digital microfluidics methods presented in this manuscript. **A.** POP assembly is an ad hoc DMF method for de-novo synthesis of DNA from synthetic oligonucleotides. **B.** M-CAD is an ad hoc DMF method for generating variants of existing DNA using predefined DNA segments from input DNA molecules (such as plasmids) and combining them in a predefined manner to generate pre-specified target molecules. primers for making modifications. **C.** Following DNA construction, we developed MIC for cloning newly constructed DNA with DMF using a cell-free technique based on single molecule PCR. While this manuscript focuses on methodology and technology development and not on integration - In the future, we envision the three methods we developed being used in a single cartridge in tandem with the output of one method serving as the input to the next method. Specifically, DNA molecules constructed from scratch using POP assembly de-novo synthesis (the output from POP) will be used as the input for M-CAD that will generate a library of molecules generated by editing the molecules generated using the principles of M-CAD assembly (defining and executing text editing operations on DNA, sharing of DNA segments between variants and combinatorial use of segment to generate a large number of output molecules from a smaller number of input molecules, The output from M-CAD and/or POP assembly can then be used to generate clones made in-vitro on the same or a different cartridge for DNA sequencing or other downstream processing operations off-cartridge.

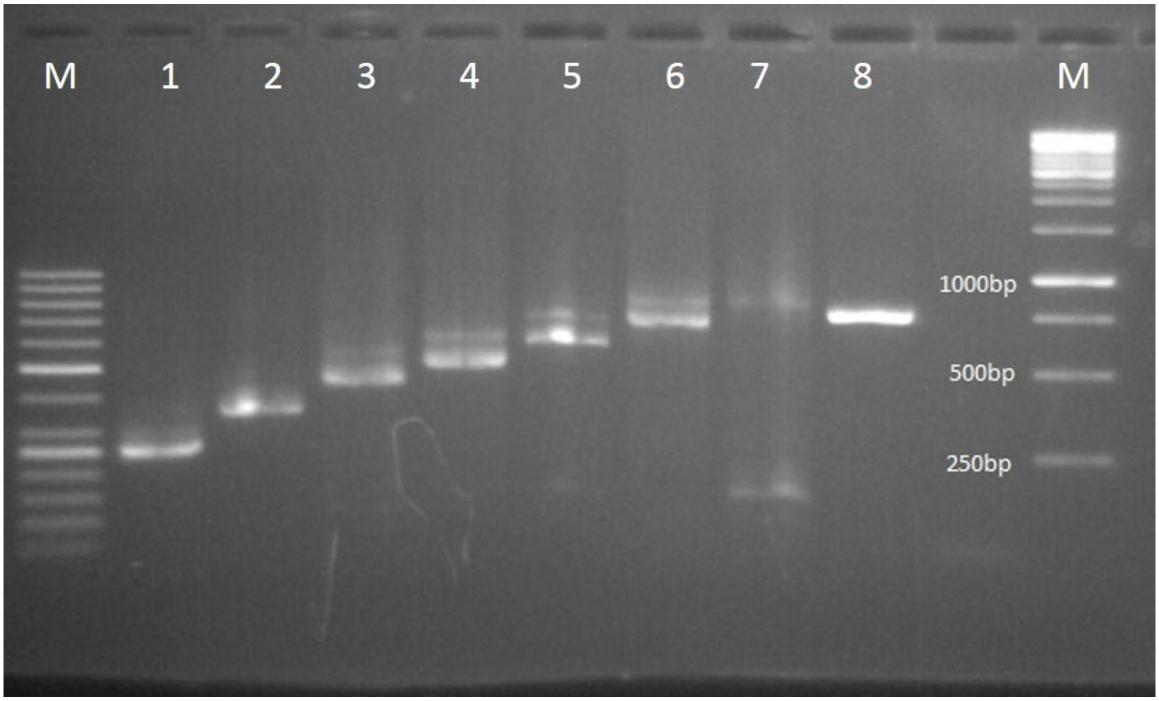

Figure S1 – Gibson assembly with increasing number (lanes 1 to 7) of DNA fragments in the assembly reaction. The last two lanes on the far right (before the marker) are the positive (lane 8) and negative (un-numbered) controls, respectively. Non-specific construction products increase with the increase in the number of reaction components, as expected, especially with the full length product (lane 7). For comparative purposes, the assembly of the same molecule was performed using POP assembly (See figures S1-S5).

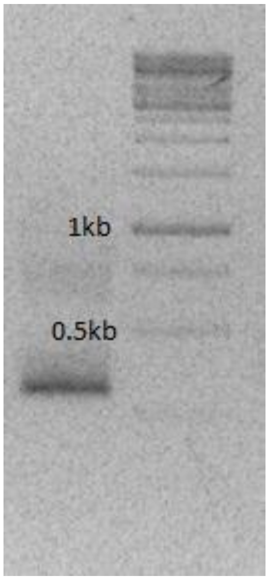

Figure S2 - POP assembly level 1. The figures shows the product of the first assembly step (left lane, AP1 in main text figure 2, enerated by AD1) in the multi-stage POP assembly procedure alongside a marker (right lane).

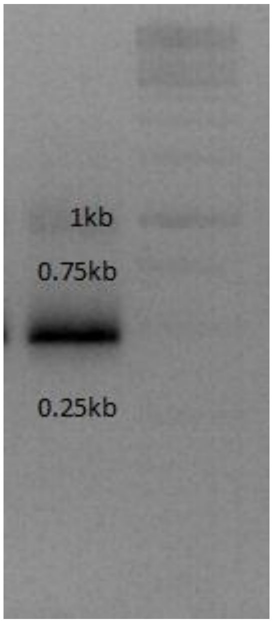

Figure S3 – POP assembly level2. The figure shows the product of the second assembly step (left lane, AP2 in main text figure 2, generated by AD2) in the multi-stage POP assembly procedure alongside a marker (right lane).

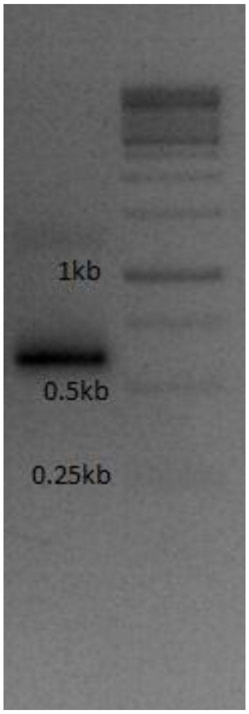

Figure S4 – POP assembly level3. The figure shows the product of the third assembly step (left lane, AP3 in main text figure 2, generated by AD3) in the multi-stage POP assembly procedure alongside a marker (right lane).

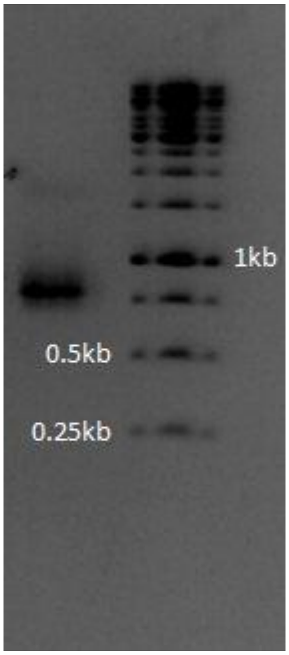

Figure S5 – POP assembly level4, producing the final target molecule of 800bp. The figure shows the product of the final assembly step (left lane, AP4 in main text figure 2, generated by AD4) in the multi-stage POP assembly procedure alongside a marker (right lane). In our experience non-specific assembly and amplification products are common in essentially all DNA assembly methods that we have tested to date. The intent in the abovementioned comparison between Gibson assembly and POP assembly was to compare the construction of an identical molecule with POP and Gibson assemblies in a DMF-independent manner. For comparative purposes the Gibson and POP reactions were both performed in tubes in order to neutralize any effect the DMF system may have and to compare the two methods with the same construction. Equimolar concentrations were used in the Gibson reaction. Both reactions show some extent of non-specific products, but the gels show that these are more abundant in the Gibson reaction compared to POP. Specifically, assembly of the correct full-length product in the Gibson reaction (lane 7 of figure S1) yielded approximately 50% correct and 50% incorrect assembly products. In contrast, while a faint non-specific band does appear on the respective gel of the full length POP assembly reaction (figure S4), the correct full-length product is by far the most abundant product in the POP reaction.

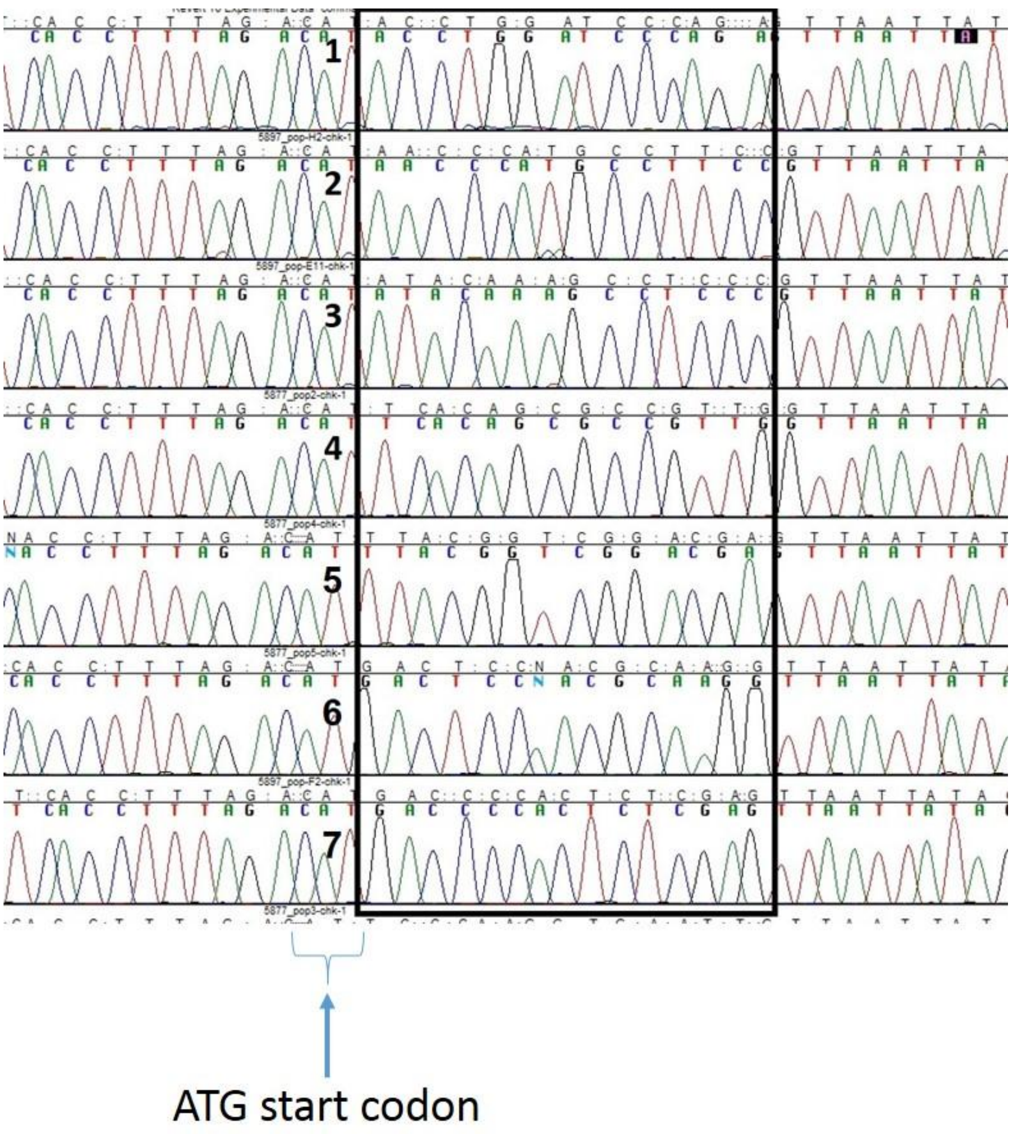

Figure S6 – Sanger sequencing chromatogram of a representative seven (rows 1-7) of the 5'UTR POP library S.cerevisiae clones. All the clones tested (clones 1-7) contained a completely different composition of nucleotides in the randomized 14 consecutive N bases region of the clones ribosome binding site, directly upstream of the ATG translation start site (marked with black rectangle), indicating that if any, a very small fraction of the POP generated library is not variable.

Construction of the Azurin library

A.

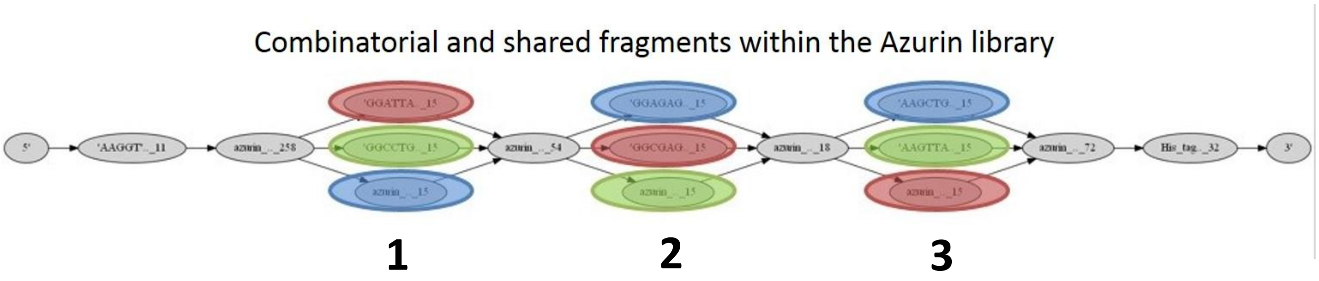

B.

Figure S7 – A. schematic representation of the structure of the Azurin library. The library has 3 variable regions (marked 1, 2 and 3). Each variable region has 3 optional segments (marked red, green and blue circles), totaling 27 possible variants (3 to the power of 3 combinations).Gray circles represents segments of the target molecules that are constant in sequence and shared between all 27 variants. The combinatorial segments (colored circles) and the shared fragments (grey circles) are PCR copied from plasmids that serve as the input fragments to the production process on the cartridge.

M-CAD overview

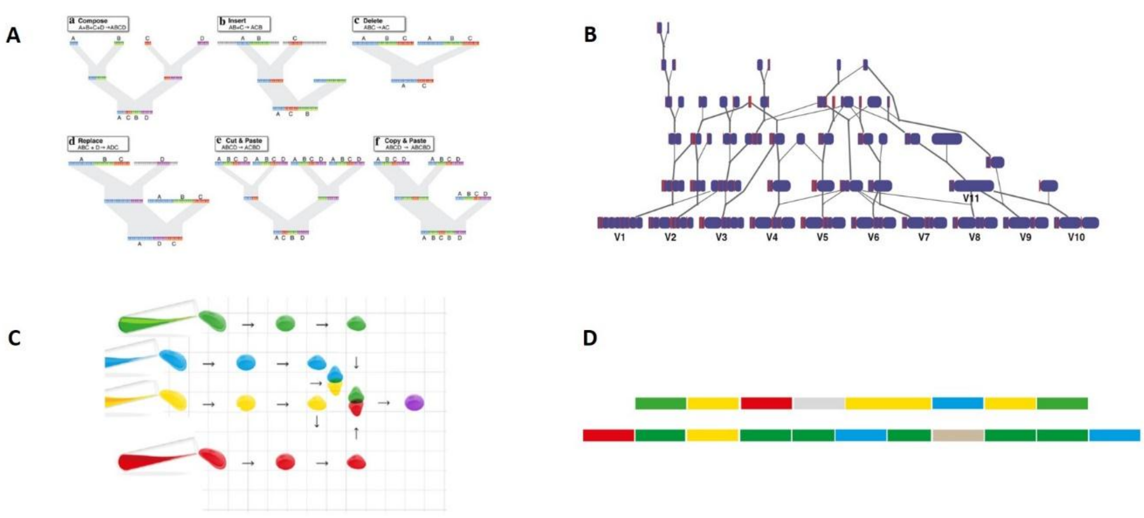

Figure S8 – Overview of M-CAD, a method that receives a set of DNA molecules as input and copies segments from them to create variants according to specification in a digital microfluidic device. **A.** M-CAD enables the implementation of the basic text editing operations on DNA molecules such as Cut, Copy, Paste, Cut and Paste, Copy and Paste etc. **B.** The text editing operations required for constructing a DNA library from a set of DNA inputs (top of the tree) are translated into a tree in which vertices are DNA molecules and edges are the editing operations (cut, paste etc..) for generating the vertices. Purple represents DNA that was originally present on the input DNA molecules and red represents synthetic DNA added during the editing process (from PCR primers for example). Vertices V1-V10 are the final 10 variant DNA molecules in this example of –CAD construction **C.** various input DNA molecules and primers are loaded onto the device and droplets from them (colored droplets) are routed on cartridge to assemble the target DNA molecules in a pre-defined manner (as determined in B). **D.** M-CAD assembly results in DNA variant molecules that are composed of predetermined combinations of DNA segments from the input molecules as well as from new synthetic DNA from synthetic oligos.

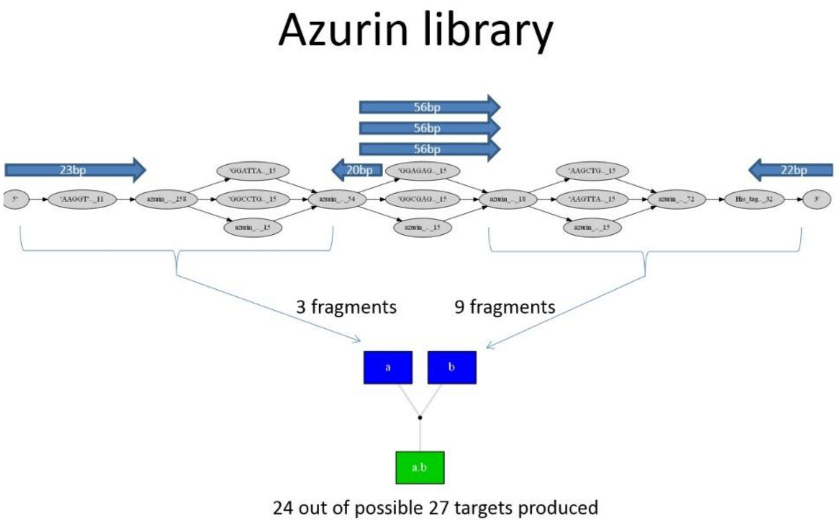

Figure S9 – Schematic representation of the structure and construction flow of the 24 variants of the Azurin library. DNA segments that form the basic building blocks for synthesizing the library are depicted in grey. Primers used to amplify these basic building blocks are depicted as blue arrows. Intermediate assembly fragments depicted as a blue node (representing 3 fragments) and a green node (representing 9 fragments)

are assembled for every possible combination to produce their product (3x9) of 27 variants.

A.

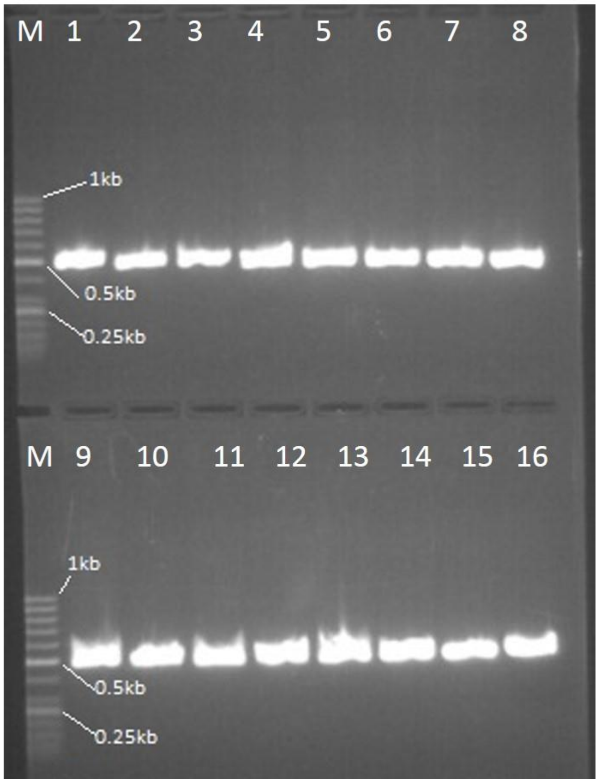

B.

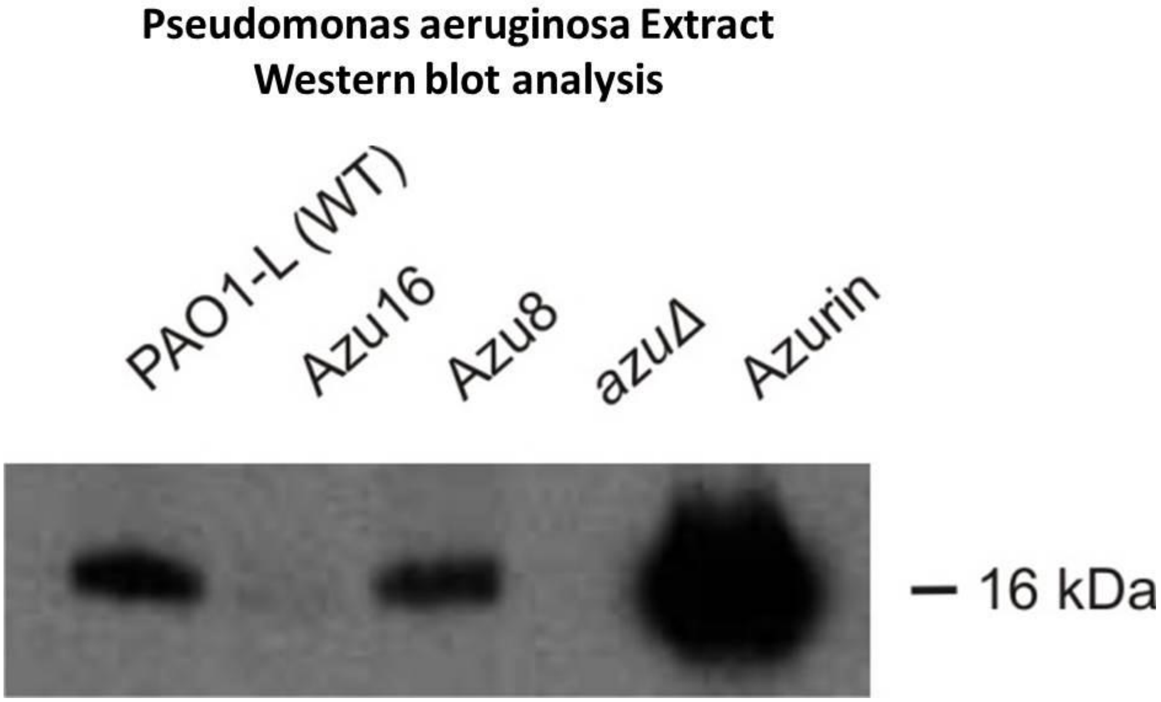

Figure S10 – a. Gel electrophoresis analysis of a representative set of 16 of the Azurin library targets shows that all constructs are of the expected size with no spurious assembly products. b. Western blot from extracts of Pseudomonas aeruginosa expressing the azu gene incubated with anti-azurin polyclonal antibodies. Cells of 16 h cultures were pelleted, suspended in SDS sample buffer and samples were run on 30% SDS-polyacrylamide gel. Lane 1: PAO1-L wild type- lane 2. !zu16 variant- lane 3. ! zu8 variant- lane 4. Δazu in frame deletion mutant- lane 5. purified azurin (0/7 µg)/. See Methods section for detailed description of methodology.

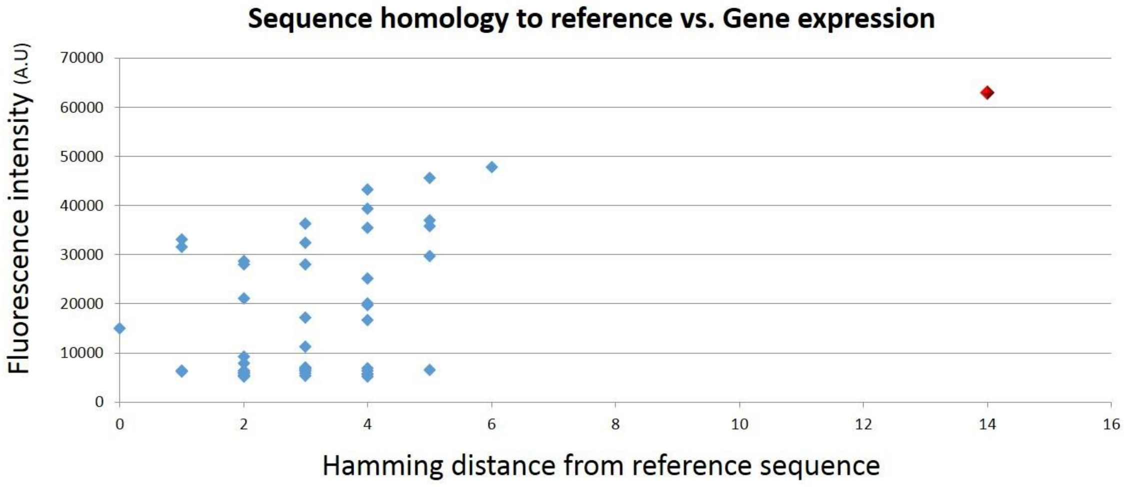

Figure S11 – the sequence of a POP library clone with a high expression RBS (red data point on the top left) was compared (using Hamming distance – a measure for sequence homology) to 40 other RBS sequences from the library. Each miss-match between two sequences reduces their Hamming score by one point (the reference has 14/14 to itself). Our 5'UTR library diversity is evident from the fact that the reference sequence has 8 or more mutations compared to all other sequences it as compared to. We performed this this analysis for all the RBSs in our library and their average Hamming score was 3.7, indicating that the RBS sequences in the library are highly diverse. As expected, sequence homology (measured in average Hamming distance) is our library is nearly identical when compared within highly expressed 5'UTRs (Hamming score 3.66) and between all sequences (Hamming score 3.7), validating previous findings that RBSs with similar gene expression are not biased towards similar sequences.

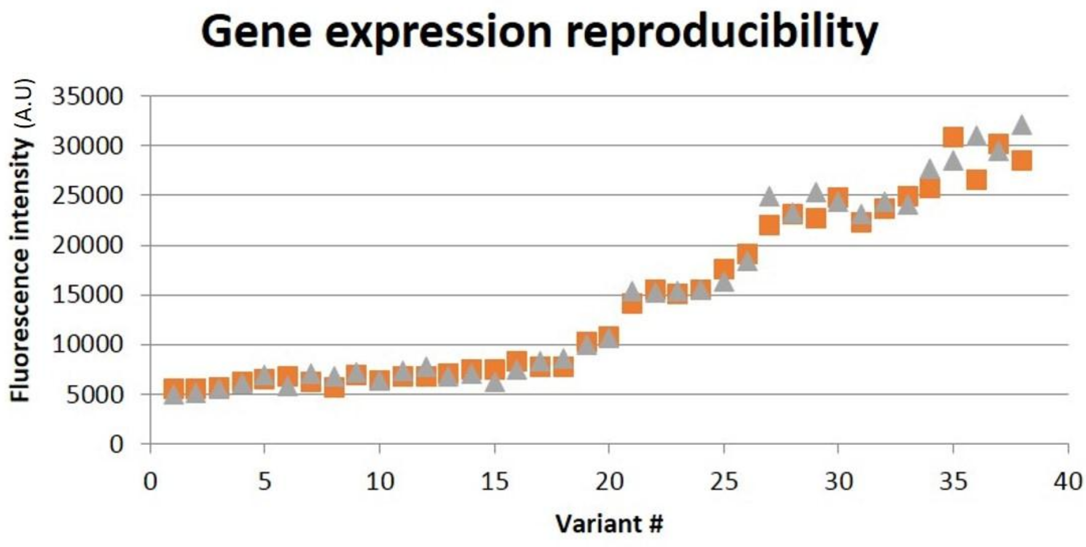

Figure S12 – Analysis of reproducibility between independent gene expression measurements of variants from the POP 5'UTR library. The gene expression from 40 POP variants (x axis) was compared in two independent measurements (grey and orange data points) and exhibited highly reproducible gene expression measurements (Y axis).

POP-Generated 5'UTR clones Sequence Analysis

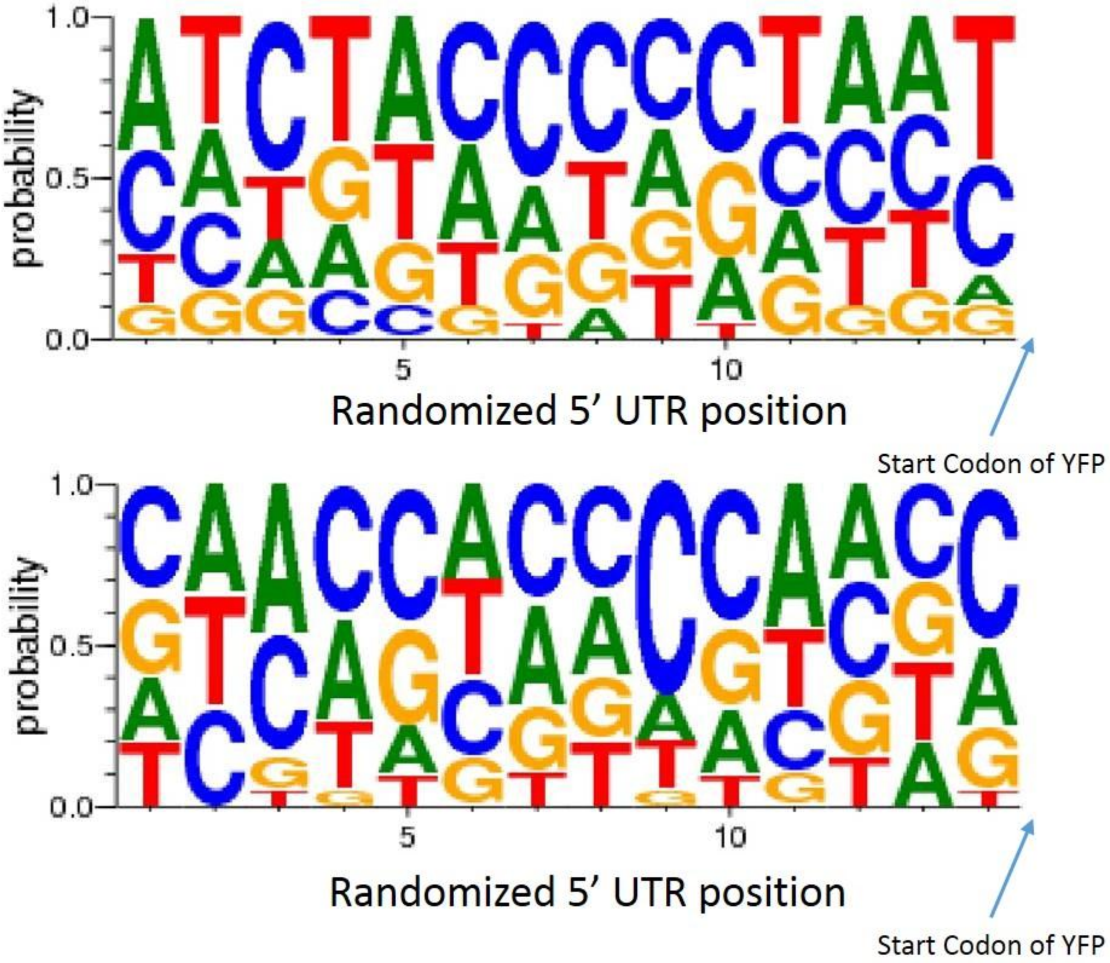

Figure S13 - Sequence analysis of the variable 5'UTR region of the POP-generated constructs. Sequences were divided into two equal groups of high (top) and low (bottom) gene expression groups according to their YFP fluorescence measurements. The library was not design to study 3'UTR's, but to validate that POP-generated construct are functional. Nevertheless, a comparative analysis of the composition of the four RNA bases within high and low expression groups showed that RBS nucleotide composition does modify gene expression, as previously shown [34]. Specifically, Uracil was the most frequent nucleotide in 8/14 positions of our 5'UTR library, compared to only 3/14 positions in low expression 5'UTRs and extreme cases of Uracil rich 5'UTRs (10/14 bases or more) were 15 fold more likely to be found in high expression compared to low expression sequence groups, which is in agreement with previous findings.

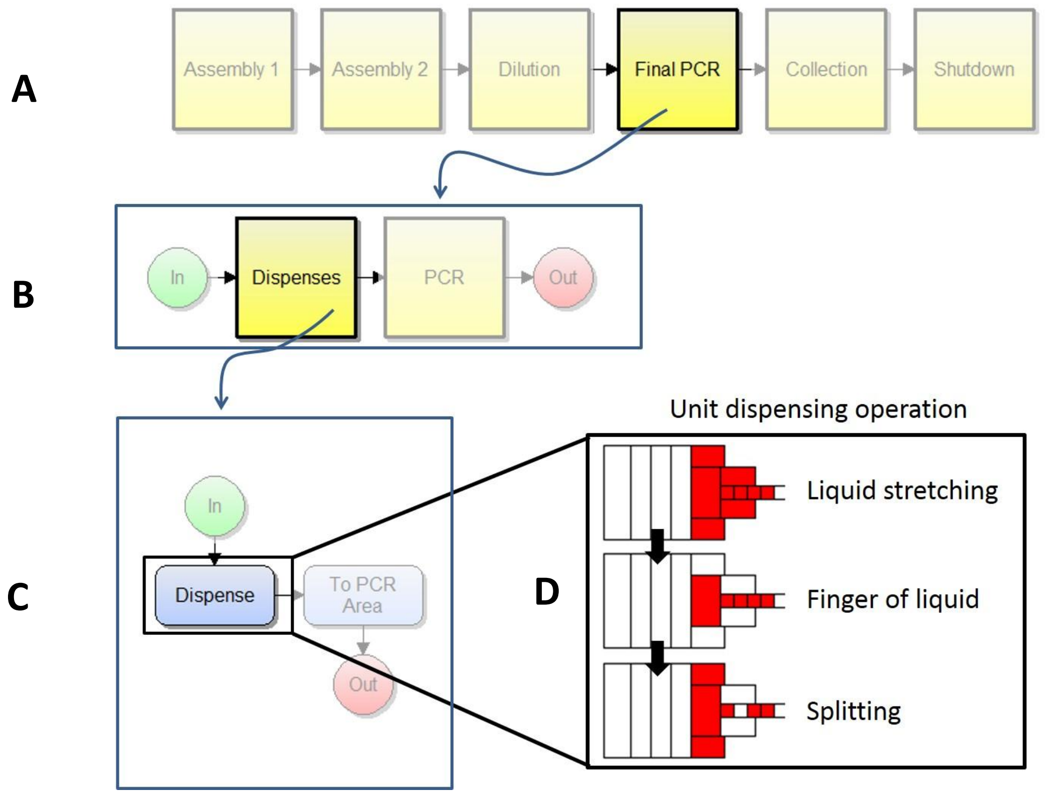

Figure S14 –Design of DMF protocols.

Successive screenshots of the graphical interface used in the DMF software. The schematics created here by the arrow and windows show a dive into each diagram showing the architecture of a typical DMF protocol. The highest level (top diagram) is a succession of individual diagrams, and the lowest level (bottom right graphical representation of some electrodes of the cartridge) represents three unit operations for droplet dispensing. DMF protocols are designed using a top-down strategy. Here we show a protocol and move in a hierarchal manner into its design process from A to D. **A.** objects of the highest hierarchal level are created for each of the major components of a protocol (depicted as six yellow boxes) such as an assembly operation (for example assembly level1 in POP) or a dilution operation (such as the dilution between POP assembly levels) or a final PCR amplification stage (such as the final PCR in MIC cloning). **B.** Zoomed in view into a box from A (which has an input and an output). In this level of design hierarchy operations inside each of these boxes are defined and are broken down to sub-processes such as the dispense operations required and the PCR cycling operations required, to name a few. **C.** In the next level of design hierarchy each of the sub-processes from B are broken down to the operations required to realize the sub-processes (such as dispense from well, move droplet from location to location). **D.** Lastly

the operations of the sub-processes specified in C are translated to atomic liquid handling operations with addresses on cartridge.

Sequences of synthetic oligos used in POP assembly on cartridge

>CPA_Fwd1

TACTTTAGGTTATGGTTTGATGTGTTTTGCTAGATACCCAGATCATATGA

AACAACATGACTTTTTCAAGTCTGCCATGCCAGAAGGTTATGTTCAAGAA

AGAA

>CPA_Fwd2

CTACTTACGGTAAATTGACCTTAAAATTTATTTGTACTACTGGTAAATTG

CCAGTTCCATGGCCAACCTTAGTCACTACTTTAGGTTATGGTTTGATGTG

TTTTG

>CPA_Fwd3

TTTTGGTTGAATTAGATGGTGATGTTAATGGTCACAAATTTTCTGTCTCC

GGTGAAGGTGAAGGTGATGCTACTTACGGTAAATTGACCTTAAAATTTAT

TTG

>CPA_Fwd4

CGGTCAACGAACTATAATTAACTAAACACTAGTACCATGTCTAAAGGTGA

AGAATTATTCACTGGTGTTGTCCCAATTTTGGTTGAATTAGATGGTGATG

TTAAT

>CPA_Rev1

CCATTCTTTTGTTTGTCAGCCATGATGTAAACATTGTGAGAGTTATAGTT

GTATTCCAATTTGTGACCTAAAATGTTACCATCTTCTTTAAAATCAATAC

>CPA_Rev2

GTATTTTGTTGATAATGGTCAGCTAATTGAACAGAACCATCTTCAATGTT

GTGTCTAATTTTGAAGTTAACTTTGATACCATTCTTTTGTTTGTCAGCCA

>CPA_Rev3

CTTTGGATAAGGCAGATTGATAGGATAAGTAATGGTTGTCTGGTAACAAG

ACTGGACCATCACCAATTGGAGTATTTTGTTGATAATGGTCAGCTAATTG

>CPA_Rev4

TCCATACCATGGGTAATACCAGCAGCAGTAACAAATTCTAACAAGACCAT

GTGGTCTCTCTTTTCGTTTGGATCTTTGGATAAGGCAGATTGATAGGATA

>CPA_Template

GCCAGAAGGTTATGTTCAAGAAAGAACTATTTTTTTCAAAGATGACGGTA

ACTACAAGACCAGAGCTGAAGTCAAGTTTGAAGGTGATACCTTAGTTAAT

AGAATCGAATTAAAAGGTATTGATTTTAAAGAAGATGGTAACATTTTAGG

>CPA-URA_Rev

GGTACTAGTGTTTAGTTAATTATAGTTCGTTGACCG

>CPA_Fwd4_KOZAK_&_smPCR

CAACACACCACCCACCCAACCGGTCAACGAACTATAATTAACNNNNNNNN

NNNNNNATGTCTAAAGGTGAAGAATTATTCACTGGTGTTGTCCCAATTTT

GGTTGAATTAGATGGTGATGTTAAT
